# Adoptive Transfer of Treg Cells Combined with Mesenchymal Stem Cells Facilitates Repopulation of Endogenous Treg Cells in a Murine Acute GVHD Model

**DOI:** 10.1371/journal.pone.0138846

**Published:** 2015-09-22

**Authors:** Eun-Sol Lee, Jung-Yeon Lim, Keon-Il Im, Nayoun Kim, Young-Sun Nam, Young-Woo Jeon, Seok-Goo Cho

**Affiliations:** 1 Institute for Translational Research and Molecular Imaging, The Catholic University of Korea College of Medicine, Seoul, Korea; 2 Laboratory of Immune Regulation, Convergent Research Consortium for Immunologic Disease, The Catholic University of Korea College of Medicine, Seoul, Korea; 3 Department of Hematology, Catholic Blood and Marrow Transplantation Center, Seoul St. Mary’s Hospital, The Catholic University of Korea College of Medicine, Seoul, Korea; Fujita Health University, School of Medicine., JAPAN

## Abstract

Therapeutic effects of combined cell therapy with mesenchymal stem cells (MSCs) and regulatory T cells (Treg cells) have recently been studied in acute graft-versus-host-disease (aGVHD) models. However, the underlying, seemingly synergistic mechanism behind combined cell therapy has not been determined. We investigated the origin of Foxp3^+^ Treg cells and interleukin 17 (IL-17^+^) cells in recipients following allogeneic bone marrow transplantation (allo-BMT) to identify the immunological effects of combined cell therapy. Treg cells were generated from eGFP-expressing C57BL/6 mice (Treg^egfp^ cells) to distinguish the transferred Treg cells; recipients were then examined at different time points after BMT. Systemic infusion of MSCs and Treg cells improved survival and GVHD scores, effectively downregulating pro-inflammatory Th×and Th17 cells. These therapeutic effects of combined cell therapy resulted in an increased Foxp3^+^ Treg cell population. Compared to single cell therapy, adoptively transferred Treg^egfp^ cells only showed prolonged survival in the combined cell therapy group on day 21 after allogeneic BMT. In addition, Foxp3^+^ Treg cells, generated endogenously from recipients, significantly increased. Significantly higher levels of Treg^egfp^ cells were also detected in aGVHD target organs in the combined cell therapy group compared to the Treg cells group. Thus, our data indicate that MSCs may induce the long-term survival of transferred Treg cells, particularly in aGVHD target organs, and may increase the repopulation of endogenous Treg cells in recipients after BMT. Together, these results support the potential of combined cell therapy using MSCs and Treg cells for preventing aGVHD.

## Introduction

Recent studies have demonstrated that therapeutic approaches based on various cells, such as mesenchymal stem cells (MSCs), natural killer cells (NK cells), natural killer T cells (NKT cells), and regulatory T cells (Treg cells), can be efficacious in improving acute graft-versus-host disease (aGVHD) complications and survival rates after allo-HSCT [1‒6]. In particular, MSCs have been widely studied in clinical HSCT to suppress the proliferation of allo-reactive T cells that are involved in aGVHD [5,7,8]. In addition, regulatory T cells (Treg cells) that are CD4+ CD25+ Foxp3+ have immunosuppressive abilities that decrease effector T cell activities [9–11]. However, current treatment using MSCs do not play a significant role in modulating or preventing aGVHD [12].

Several studies have demonstrated that the infusion of MSCs can–relatively–control Th1-mediated responses, but does not inhibit Th17-mediated conditions, such as autoimmune arthritis [13,14]. Treg cells are also unstable, with the potential to convert to inflammatory Th17 cells in Th1 responses in autoimmune conditions [15–17]. However, it has recently been demonstrated that interactions with MSC can induce Treg cells in various *in vitro* and *in vivo* models [18–20]. MSC-induced Treg cell formation involves several factors, including transforming growth factor beta 1 (TGF-β) and prostaglandin E2 (PGE2). In addition, co-cultures of peripheral blood mononuclear cells (PBMCs) with MSCs generated powerful regulatory CD4^+^ and/or CD8^+^ lymphocytes [19–22]. These reports suggest that MSCs may be helpful in generating and maintaining Treg cells stably in aGVHD models. Furthermore, combined cell therapy using MSCs and Treg cells may be helpful in alleviating aGVHD.

Given this background, we previously demonstrated that combined cell therapy with MSCs and Treg cells induced long-term survival in a aGVHD model and regulated Th1/Th17 cells, and Foxp3^+^ Treg cells, reciprocally in recipients [23]. In addition, we identified various therapeutic effects in mixed chimerism and skin allograft transplantation [24,25]. However, the underlying immunological mechanisms that occur in recipients have not been fully explained.

Satisfactory therapeutic outcomes in adoptive cell therapy depend on whether the adoptively transferred cells remain in recipients over a long period of time without conversion to other cell types. Thus, we demonstrated combined cell therapy using *ex vivo*-expanded Treg cells from eGFP-expressing C57BL/6 mice (Treg^egfp^ cells). We distinguished the adoptively transferred Treg^egfp^ cells and repopulating endogenous Treg cells following co-administration of Treg^egfp^ cells with MSCs in recipients after BMT. We demonstrate the functional properties of MSCs to maintain and generate Treg cells *in vivo*, and also provide a potential strategy for treating aGVHD through combined cell therapy in aGVHD.

## Materials and Methods

### Mice

BALB/c (H-2d) mice (8–10 weeks old) were purchased from OrientBio (Sungnam, Korea). C57BL/6-tg(CAG-EGFP; H-2^b^) mice were purchased from Japan SLC (Shizuoka, Japan). The mice were maintained under specific pathogen-free conditions in an animal facility with controlled humidity (55±5%), light (12/12 h light/dark), and temperature (22±1°C). The air in the facility was passed through a HEPA filter system, designed to exclude bacteria and viruses. Animals were fed mouse chow and tap water *ad libitum*. For blood collection, mice were anesthetized with 2.5% isoflurane in oxygen, and sacrificed by exposure to CO_2_. The animal care and euthanasia protocols used in this study were approved by the Animal Care and Use Committees at Korea University and the Catholic University of Korea, College of Medicine (Permit number: 2013-0073-01).

### Treg generation

To obtain Treg cells, isolated CD4+ T cells from C57BL/6-tg(CAG-EGFP) mice were cultured with plate-bound anti-CD3 (1 μg/mL; BD PharMingen, CA, USA), soluble anti-CD28 (1 μg/mL; Biolegend, San Diego, CA, USA), human recombinant transforming growth factor beta (TGF-β; 5 ng/mL; PeproTech, London, UK), and RA (100 nM; Sigma-Aldrich, St. Louis, MO, USA) for 3 days. The expanded induced Treg cells were sorted by flow cytometry to obtain a > 96% pure CD4^+^ CD25^+^ Foxp3^+^ population.

### Isolation and culture of MSCs

C57BL/6 BM cells were collected by flushing femurs and tibias with Dulbecco’s Modified Eagle’s Medium (Gibco, Carlsbad, CA, USA) containing 2 mM L-glutamine (Gibco), 1% antibiotics (penicillin [10 U/mL]-streptomycin [10 g/mL]; Gibco), and 15% heat-inactivated fetal bovine serum (FBS) with endotoxin levels ≤ 5 EU/mL and hemoglobin levels ≤ 10 mg/dL (Gibco). Cell immunophenotypes were persistently positive for Sca-1, CD44, and CD29, but were negative for c-Kit, CD11b, and CD34 after more than 15 passages using the antibodies described below, consistent with previous reports [26].

### Bone Marrow Transplantation and aGVHD Induction

Recipient (BALB/c, H-2^d^) mice were exposed to a 800 cGy dose of radiation from a Mevatron MXE-2 instrument (Siemens, New York, NY, USA), with a focus on skin distance of 100 cm and a rate of 70 cGy/min. Recipient mice were then injected intravenously (IV) with 5×10^6^ BMCs and 5×10^6^ spleen cells from donor mice (C57BL/6, H-2^b^). Survival after bone marrow transplantation (BMT) was monitored daily, and the degree of clinical aGVHD was assessed weekly using a scoring system that summed changes in five clinical parameters: weight loss, posture, activity, fur texture, and skin integrity. Animals with scores > 7 were considered moribund and were euthanized 50 days after transplantation.

### Combined cell therapy of MSCs and Treg cells controlled aGVHD

Mice were injected i.p. with 1×106 MSCs, and i.v. with 2×106 Treg cells or 1×106 MSCs plus 2×106 Treg cells twice weekly after BMT (BMT + day 0, 4). Control mice received i.v. injections of an equal volume of phosphate-buffered saline (PBS; Gibco) at the same time points.

### Flow cytometric analysis

Mononuclear cells were immunostained with various combinations of the following fluorescence-conjugated antibodies: CD25-APC (eBioscience, San Diego, CA, USA), CD4-Paciffic blue (eBioscience), Foxp3-PE (eBioscience), H-2^d^-percp-cy5.5 (eBioscience), IL-17-PE (eBioscience), Foxp3-APC (eBioscience), IFN-γ-APC (eBioscience), IL-4-PE (BD PharMingen), IL-17-FITC (eBioscience), H-2^b^-FITC (eBioscience), IL-6-PE (Biolegend), and TNF-α-APC (BD). Before intracellular cytokine staining, cells were stimulated in culture medium containing phorbol myristate acetate (25 ng/mL; Sigma-Aldrich), ionomycin (250 ng/mL; Sigma-Aldrich), or monensin (GolgiStop, 1 μL/mL; BD PharMingen) in a 5% CO_2_, 37°C incubator for 4 h. Intracellular staining was performed using an intracellular staining kit (eBioscience) according to the manufacturer’s protocol. Flow cytometry was performed on a FACSCalibur flow cytometer (BD PharMingen) with the FlowJo software (TreeStar, Ashland, OR, USA).

### Western blotting

Spleen tissues were collected from recipients at 10 days after allo-BMT. Protein samples were separated by SDS gel electrophoresis, and transferred to a nitrocellulose membrane (Amersham Pharmacia Biotech, Buckinghamshire, UK). Membranes were stained with primary antibodies against signal transducer and activator of transcription (STAT) 1, STAT3 (all from Cell Signaling, Danvers, MA, USA), and β-actin. Then, horseradish peroxidase (HRP)-conjugated secondary antibody was added. After washing with Tris-buffered saline plus Tween-20 (TTBS), the protein bands were detected using an enhanced chemiluminescence (ECL) detection kit and Hyperfilm-ECL reagents (Amersham Pharmacia Biotech, Piscataway, NJ, USA).

### Analysis of gene expression using real-time quantitative PCR

Total RNA was extracted using the TRIzol reagent (Invitrogen). Total RNA (2 μg) was reverse transcribed at 50°C for 2 min, followed by 60°C for 30 min. Quantitative PCR was performed using a FastStart DNA Master SYBR Green I kit and a LightCycler 480 detection system (Roche, Meylan, France), as specified by the manufacturer. The crossing point (Cp) was defined as the maximum of the second derivative of the fluorescence curve. Negative controls contained all elements of the reaction mixture, except the template DNA. For quantification, the relative mRNA expression of specific genes was obtained by the ΔΔCt method, using β-actin for normalization. The following gene-specific primers (5’→3’) were used: β-actin (forward, GAA ATC GTG CGT GAC ATC AAA G and reverse, TGT AGT TTC ATG GAT GCC ACA G), Foxp3 (forward, GGC CCT TCT CCA GGA CAG A and reverse GGC GCT GAT CAT TGG GTT GT), eGFP (forward, TGA ACC GCA TCG AGC TGA AGG G and reverse, TCC AGC AGG ATG TGA TCG C), RORγt (forward, TGT CCT GGG CTA CCC TAC TG and reverse, GTG CAG GAG TAG GCC ACA TT), and SOCS3 (forward, CGC CTC AAG ACC TTC AGC TC and reverse, CTG ATC CAG GAA CTC CCG AA).

### Histopathology of aGVHD

Mice were sacrificed on day 21 after BMT for blinded histopathological analysis of aGVHD targets (liver and small intestine). Organs were harvested, cryoembedded, and then sectioned. Tissue sections were fixed in 10% buffered formalin (Sigma-Aldrich) and stained with hematoxylin (Sigma-Aldrich) and eosin Y 1% solution (Muto Pure Chemical Co., Ltd., Tokyo, Japan) for histological examination. The scoring system for each parameter was 0 for normal, 0.5 for focal and rare, 1 for focal and mild, 2 for diffuse and mild, 3 for diffuse and moderate, and 4 for diffuse and severe, in accordance with previously reported aGVHD histology [27].

### Immunohistochemistry of aGVHD target organs

Paraffin sections (5 μm thick) were deparaffinized in xylene followed by hydration through graded ethanols. Antigen retrieval was performed by heating specimens at 92–95°C for 10 min with Universal Antigen Retrieval Agent (CTS015; R&D Systems, Minneapolis, MN). Endogenous peroxidase was blocked by incubating sections with 0.3% hydrogen peroxide for 15 min, and the non-specific binding sites were blocked in PBS with 1% horse serum for 30 min. Sections were covered with primary antibody, and the slides were incubated in a moist chamber overnight at 4°C (Invitrogen anti-GFP rabbit IgG, A11122). After three washes in PBS, sections were incubated with secondary antibody (FITC-conjugated goat anti-rabbit IgG #115922) at 1:200 in incubating solution (1% BSA, 1% normal donkey serum, 0.3% Triton X-100 and 0.01% sodium azide in PBS) for 1 h at room temperature. After three washes in PBS, sections were incubated with DAPI for 10 min at room temperature, before rinsing once with PBS, and mounting in fluorescence mounting medium (Dako #S3023). Fluorescence images were acquired using an LSM 510 confocal microscope (Carl Zeiss, Jena, Germany).

### Statistical Analysis

Statistical analyses were performed using Graphpad Prism (ver. 5.01). Comparisons between groups were analyzed statistically using the Kruskal-Wallis test. Pair-wise group comparisons used the Mann-Whitney U-test, and *p* values were adjusted for multiple comparisons using Bonferroni’s method to determine the statistical significance of these comparisons. A *p* value < 0.05 was considered statistically significant.

## Results

### Immunophenotypes of culture-expanded MSCs and Treg cells

**Fig 1:**
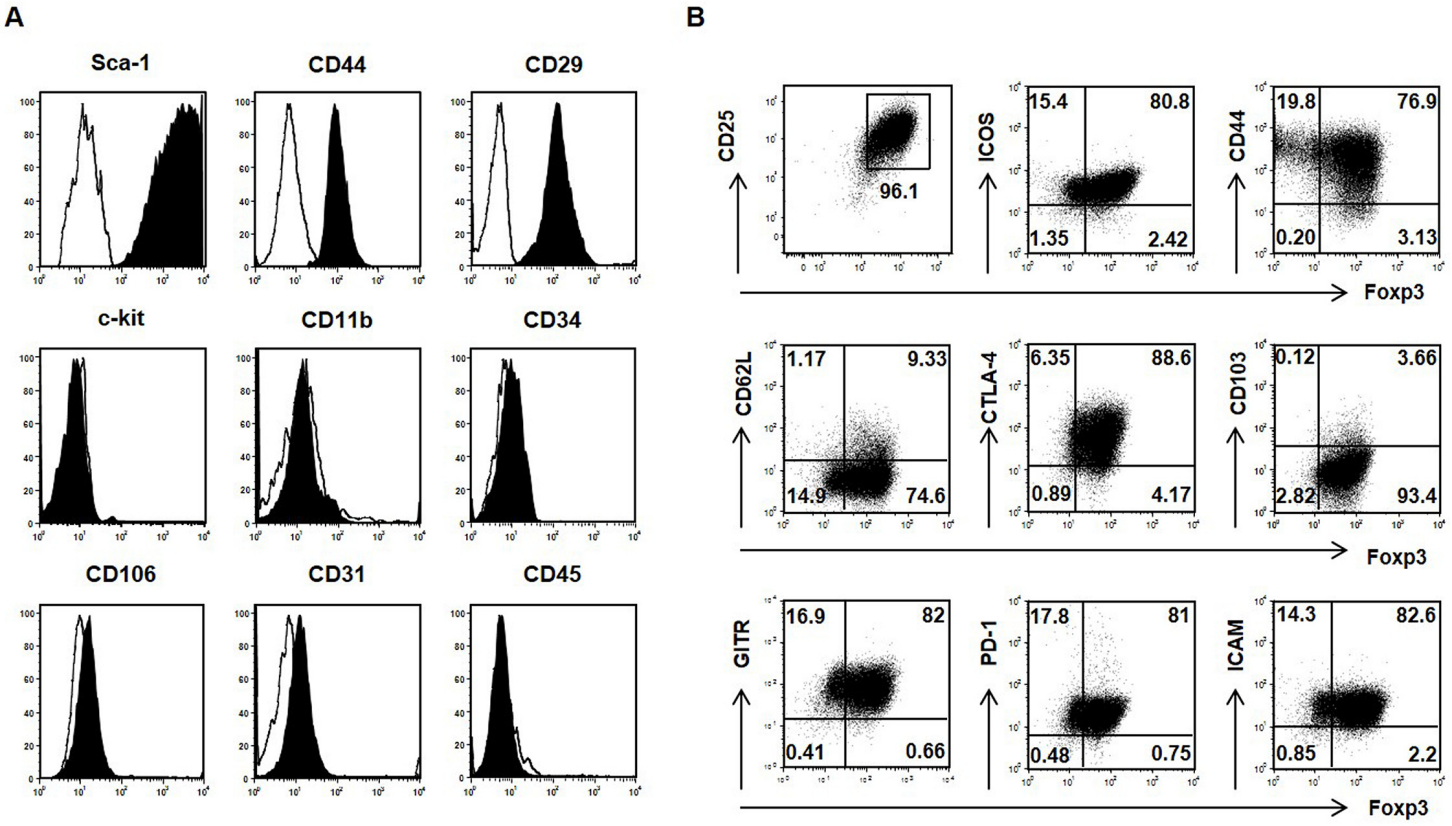
Characterization of donor-derived MSCs and Treg cells. (A) Culture-expanded MSCs were distinguishable from hematopoietic cells by being positive for Sca-1, CD44, and CD29, but were negative for the cell surface markers c-kit, CD11b, CD34, CD106, CD45, and CD31. (B) The purity of Treg cells was greater than 96% by flow cytometry. Treg cells were analyzed by FACS for intracellular Foxp3, CTLA-4, PD-1, GITR, ICAM-1, and ICOS, and surface expression of the indicated cellular markers CD44, CD62L, and CD103 in the gated CD4^+^ T cell population.

To characterize culture-expanded MSCs and Treg cells from C57BL/6 mice, surface protein expression of MSCs was examined using flow cytometry at the 10th–15th passage. MSCs showed a typical fibroblast-like morphology, and were uniformly positive for Sca-1, CD44, and CD29, but were negative for c-Kit, CD11b, CD34, CD106, CD45, and CD 31 (Fig 1A) [26]. CD4^+^CD25^+^Foxp3^+^ Tregs showed >96% purity on flow cytometry and positive surface staining for several phenotypic Treg markers, including CD44, glucocorticoid-induced tumor necrosis factor receptor (GITR), intercellular adhesion molecule-1 (ICAM-1), inducible costimulator (ICOS), and programmed death-1 (PD-1). They also showed weak positive surface staining for CD62L and CD103 (Fig 1B).

### Donor-derived MSCs and Treg cells improve clinical outcomes in a murine aGVHD model

**Fig 2:**
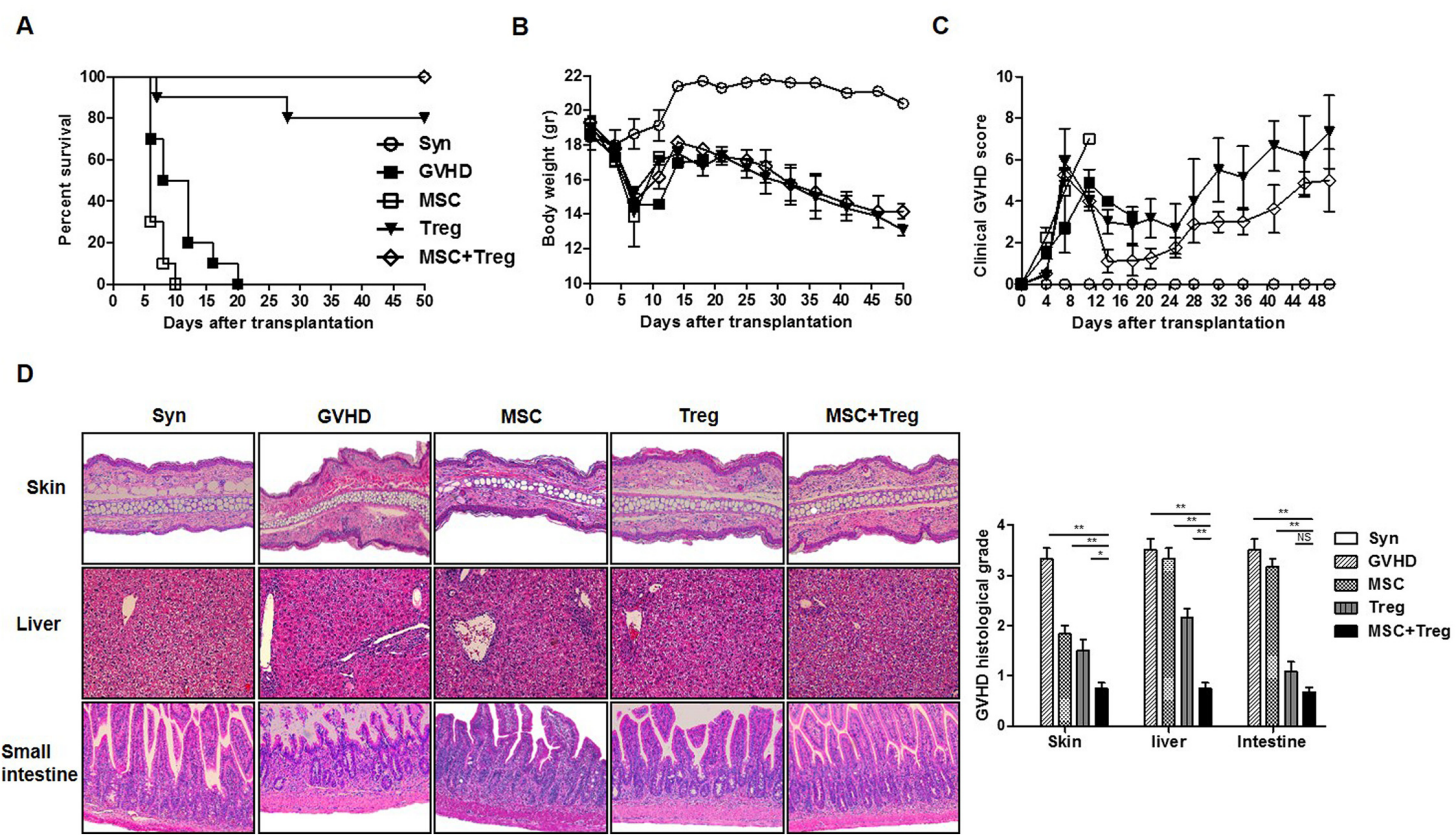
Improvement in aGVHD severity with donor-derived MSCs and Treg cells. Recipient mice (BALB/c, H-2^d^) were irradiated with 800 cGy and injected intravenously (i.v.) with 5×10^6^ BMCs and 5×10^6^ spleen cells from donor mice (C57BL/6, H-2^b^) and donor-derived MSCs (1×10^6^) and C57BL/6 background eGFP mice-derived Treg cells (2×10^6^) on days 0 and 4, and mice were evaluated at different time points after BMT (*n* = 10). (A) Increase in survival rate in aGVHD mice model after co-administration of MSCs and Treg cells in allogeneic transplantation. (B–C) The degree of clinical aGVHD was assessed weekly using a scoring system that summed changes in five clinical parameters: weight loss, posture, activity, fur texture, and skin integrity. (D) Histological scores were evaluated in aGVHD target organs: skin (200×), liver (200×), and small intestine (200×). Data are shown as the mean ± SEM. The results are representative of three independent experiments. *P* values were determined using Student’s *t*-test. * *p* < 0.05, ** *p* < 0.01.

aGVHD is caused by donor-derived T cells that attack recipient tissues after allo-HSCT from a major histocompatibility complex (MHC)-unrelated donor. Recipients (BALB/c, H-2d) were lethally irradiated and received BM cells with spleen cells from donor mice (C57BL/6, H-2d). At days 0 and 4 after BMT, recipients were administered MSCs (1×106) and Treg^egfp^ cells (2×106) from donor-derived mice (H-2b). Mice were evaluated at different time points after BMT. The mice were monitored for survival, body weight, and clinical aGVHD scores. Survival was 80% in the Treg cells group and 100% in the combined cell therapy group (Fig 2A). Body weights were not significantly different compared to the Treg cell group (Fig 2B), and aGVHD scores were improved in the combined cell therapy group compared to the single-therapy groups (Fig 2C). Interestingly, MSCs alone had lower survival and clinical scores. Histological analyses of aGVHD target organs also showed improvements in the combined cell therapy group (Fig 2D). These data suggest that combined cell therapy with donor-derived MSCs and Treg cells was effective for aGVHD.

### Co-administration of donor-derived MSCs and Treg cells downregulate Th1 responses in an aGVHD model

**Fig 3:**
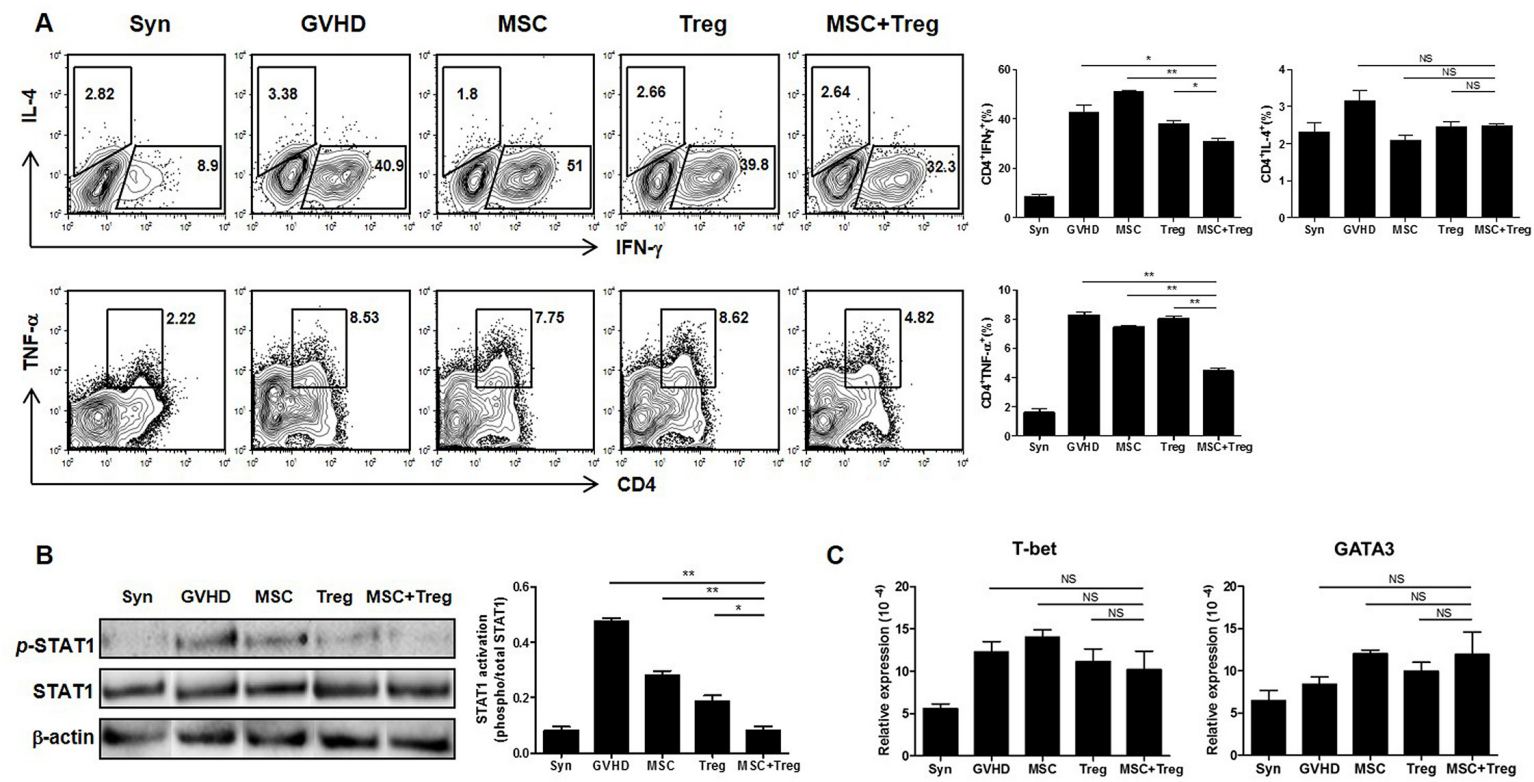
Downregulation of Th1 response by co-infusion of donor-derived MSCs and Treg cells in aGVHD model. (A) Th1 and Th2 cells were examined by flow cytometry at 12 days after BMT. IFN-γ and TNF-α decreased in the combined cell therapy group compared to the other single groups, whereas IL-4 expression showed no significant difference among the groups. (*n* = 6). (B) Expression of phosphorylated-(p) STAT1 was significantly downregulated in the combined cell therapy group versus each single group, as determined by Western blot analysis. (C) T-bet and GATA3 mRNA levels were examined by real-time PCR. There were no group had differences in T-bet or GATA3 levels. Data are shown as means ± SEM. The results are representative of three independent experiments. *P* values were determined using Student’s *t*-test. * *p* < 0.05, ** *p* < 0.01

Th1 cells play an important role in aGVHD pathogenesis, whereas Th2 cells reduce aGVHD [28,29]. We examined the improvement in aGVHD using donor-derived MSCs and Treg cells, and performed an *ex vivo* immunological analysis. We observed the percentage of Th1 and Th2 cells in recipient spleen at 12 days after BMT. The percentages of IFN-γ and TNF-α secreted by Th1 cells decreased in the combined cell therapy group versus MSCs or Treg cells alone. IL-4, which is secreted by Th2 cells, showed no significant difference among the groups (Fig 3A). In addition, the expression of phosphorylated-(p) signal transducers and activators of transcription 1 (STAT1) was significantly downregulated in the combined cell therapy group (Fig 3B). The mRNA expression of T-box transcription factor TBX21 (T-bet) and trans-acting T-cell-specific transcription factor GATA-3 (GATA3), transcription factors of Th1 and Th2 cells, were measured in recipient spleen using real-time PCR. The mRNA levels of T-bet were decreased in the combined cell therapy group, whereas GATA-3 was not significantly different among groups. There were no groups had significant group differences in T-bet or GATA3 (Fig 3C). It has been suggested that Th1 expression was effectively downregulated by combined cell therapy in the aGVHD model.

### Effective downregulation of Th17 cells by co-infusion of MSCs and Treg cells

**Fig 4:**
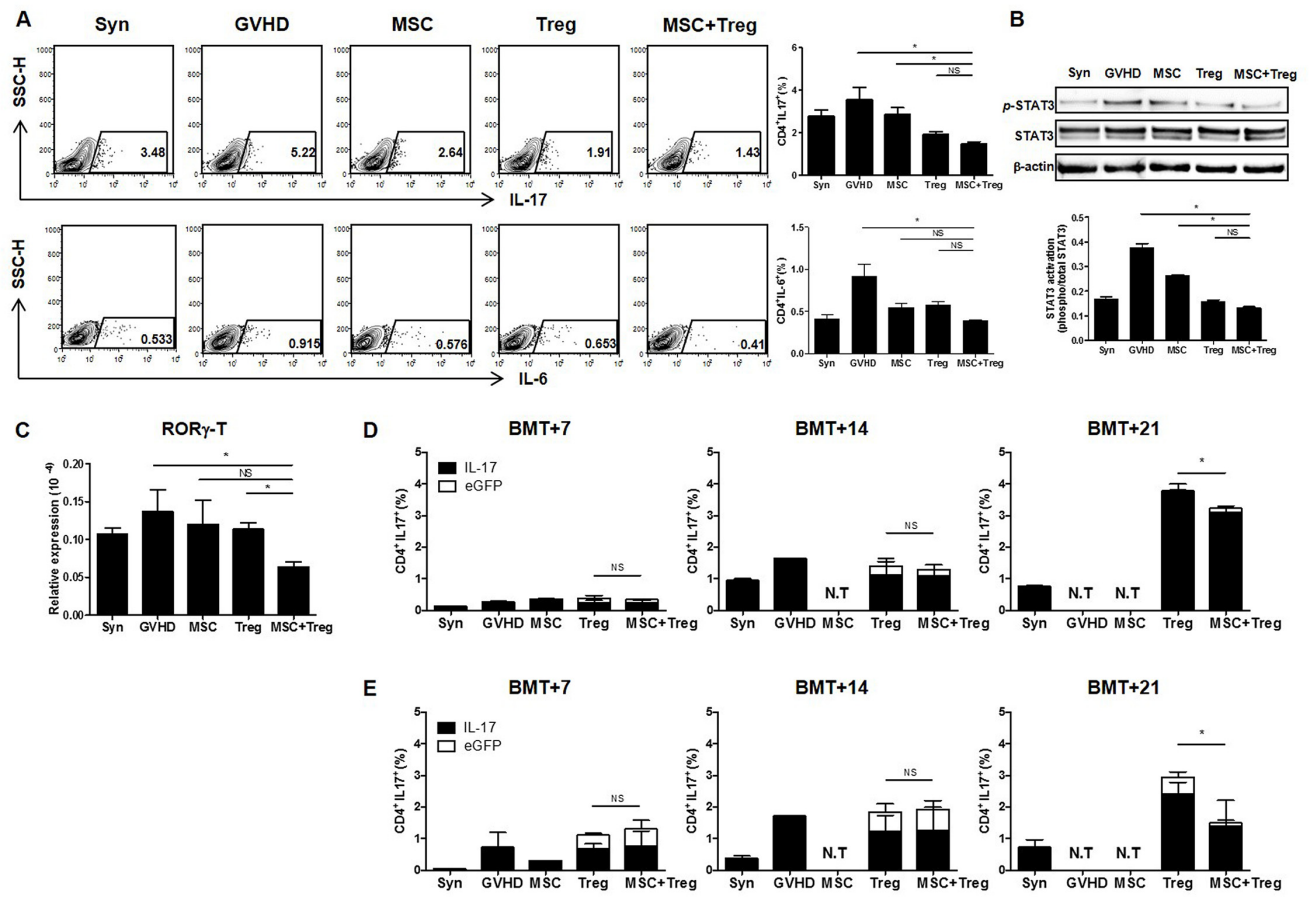
Reduction of IL-17 after co-administration of donor-derived MSCs and Treg cells. Recipient mice were evaluated at different time points after BMT. (A) The percentage of CD4^+^ IL-17^+^ cells and CD4^+^ IL-6^+^ cells were identified by flow cytometry at 12 days after BMT (*n* = 6). (B) Expression of p-STAT3 was significantly downregulated in the combined cell therapy group compared to the single groups, as determined by Western blotting. (C) RORγ-t mRNA levels were significantly decreased in the combined cell therapy group versus the Treg cells single group using real-time PCR. The percentage of IL-17^+^ cells detected in the (D) spleen and (E) blood by flow cytometry at 7, 14, and 21 days after BMT. CD4^+^ IL-17^+^ eGFP^+^ cells (white bar) were detected as a small proportion of CD4^+^ IL-17^+^cells. Data are shown as means ± SEM. The results are representative of three independent experiments. *P* values were determined by Student’s *t*-test. * *p* < 0.05.

The functional imbalance of Treg cells and Th17 cells is key in severe aGVHD; several reports have demonstrated instability of Treg cells that convert into Th17 cells under Th1 conditions [15–17]. We identified IL-17 and IL-6 expression in recipient spleen. IL-17 cells, within CD4^+^ T cells, were significantly reduced in the combined cell therapy group, compared to MSCs alone or the GVHD group, 12 days after BMT. While IL-17 levels in the combined cell therapy group were reduced compared to those of the Treg group, there was no significant difference. A trend toward a reduction of IL-6 levels was observed in the combined cell therapy group; however, there was only a significant difference between the GVHD and combined cell therapy groups (Fig 4A). In addition, STAT3, which plays a role in downregulating Foxp3 in aGVHD, was decreased in the combined cell therapy group (Fig 4B). In addition, we identified that the transcription factor, RAR-related orphan receptor gamma (RORγt), which binds IL-17 in naïve CD4^+^ T helper cells, was significantly decreased in the combined cell therapy group compared to the Treg cell group (Fig 4C).

We identified the percentage of IL-17^+^ cells within CD4^+^ T cells in the recipient spleen and blood at three time points after BMT. We used *ex vivo*-expanded Treg cells from C57BL/6 eGFP^+^ (H-2b) background mice that had > 96% purity on flow cytometry. Recipients were administered donor-derived MSCs (1x10^6^) and eGFP+ Treg cells (2x10^6^) on days 0 and 4 after allo-BMT. We examined eGFP^+^IL-17^+^ cells of CD4^+^ T cells that represent IL-17^+^ cells converted from infused eGFP^+^ Treg cells after BMT. IL-17 expression was detected in < 0.5% of CD4^+^ T cells in the spleen, and eGFP^+^ IL-17^+^ cells were barely detected on day 7. IL-17^+^ cells increased slowly by day 21; however, eGFP^+^ IL-17^+^ cells were still a small proportion of total CD4^+^ T cells (Fig 4D). Moreover, the detected IL-17^+^ cells were completely of donor (H-2b) origin, endogenously generated in the recipient after BMT (data not shown). Similar to the spleen, IL-17^+^ cells were detected at a maximum percentage of 3% of CD4^+^ T cells in blood. In addition, IL-17^+^ cells were downregulated in the combined cell therapy group compared to the Treg cells single group (Fig 4E). These results suggest that MSCs could aid in maintaining adoptively transferred Treg cells stably in recipients.

### MSCs contribute to the maintenance of adoptive transferred Treg cells, and help in the repopulation of endogenous Treg cells in recipients

We examined the origin of Foxp3+ Treg cells that increased in recipients after BMT. We used *ex vivo*-expanded Treg cells from C57BL/6 background eGFP+ (H-2b) mice that showed > 96% purity on flow cytometry. Recipients were administered donor-derived MSCs (1×106) and Treg^egfp^ cells (2×106) on days 0 and 4 after allo-BMT.

**Fig 5:**
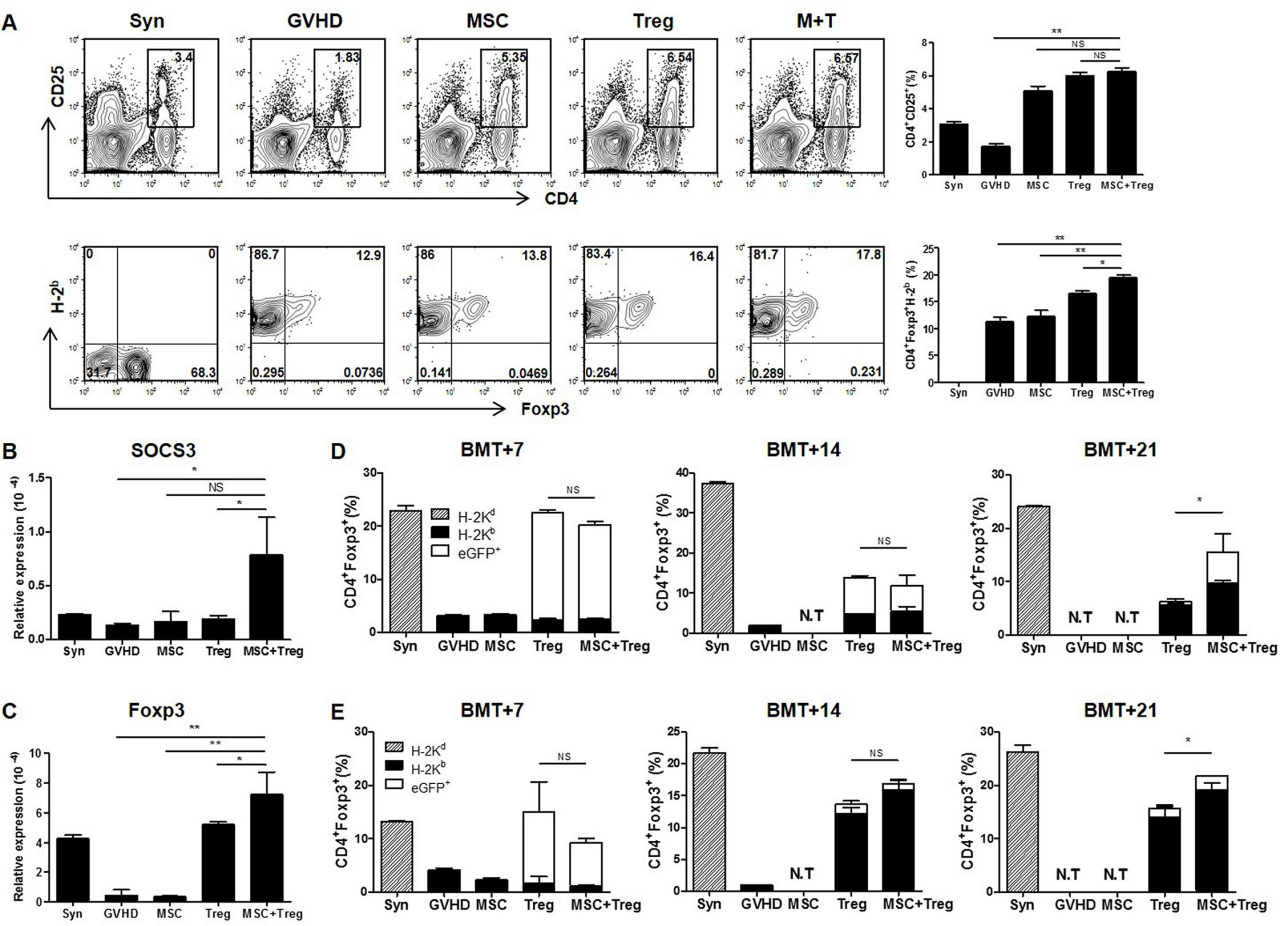
Identification of the origin of CD4^+^ Foxp3^+^ Treg cells that increased in recipients using *ex vivo*-expanded Treg cells from eGFP^+^ (H-2^b^) mice. Recipients (BALB/c, H-2^d^) were administered donor-derived MSCs (1×10^6^) and eGFP+ Treg cells (2×10^6^) on days 0 and 4, and were sacrificed on days 7, 14, and 21 after BMT (*n* = 6). (A) The gated CD4^+^ CD25^+^ populations of H-2K^b+^ Foxp3^+^ cells were examined at 12 days after BMT by flow cytometry. (B–C) Expression of SOCS3 and Foxp3 was significantly upregulated in the combined cell therapy group at 12 days after BMT by real-time PCR. Animals in the GVHD and MSCs groups all died within 20 days after BMT. CD4^+^ Foxp3^+^ Treg cells were measured in recipients’ (D) spleen and (E) blood by flow cytometry at days 7, 14, and 21 after BMT. H-2b cells indicate endogenous donor-origin CD4^+^ Foxp3^+^ Treg cells (black bar), and eGFP^+^ cells indicate adoptive transferred Treg cells (white bar). Host-origin (H-2d) cells were not detected. Data are shown as means ± SEM. The results are representative of three independent experiments. *P* values were determined using a Student’s *t*-test. * *p* < 0.05, ** *p* < 0.01.

CD4^+^ CD25^+^ cells from recipients generally increased in the groups that received cells for therapy, except the GVHD group. Furthermore, Foxp3^+^ cells were increased in the combined cell therapy group, compared to the other groups (Fig 5A). In addition, we identified several transcription factors, suppressor of cytokine signaling 3 (SOCS3) and forkhead box protein 3 (Foxp3), which are important in evaluating aGVHD, and which significantly increased in the combined cell therapy group compared to the Treg cells alone group (Fig 5B and 5C).

We identified the percentage of eGFP^+^ cells in the Foxp3^+^ cells in the spleen and blood of the recipient by flow cytometry. Foxp3+ Treg cells were mostly of eGFP+ origin in both the Treg cells single group and the combined cell therapy group on day 7. On day 14, the eGFP+ cells in the Foxp3+ Treg cells showed a lower percentage than on day 7, but were still maintained in both the Treg cells single group and combined cell therapy group. However, eGFP+ cells were still only detected at a high level in the combined cell therapy group on day 21, but not in the Treg cells single group (Fig 5D). Also notable was the fact that donor-derived (H-2b) Foxp3+ Treg cells significantly increased in the combined cell therapy group. These results suggest that combined therapy with MSCs and Treg cells increased the repopulation of Foxp3^+^ Treg cells from the recipient after BMT.

Likewise, Foxp3^+^ Treg cells were mostly of eGFP origin in recipient blood on day 7. However, endogenous Treg cells (H-2b), generated in the recipient, were increased in both the Treg cells and combined cell therapy groups. The Treg cells were increased in the combined cell therapy group compared to the Treg cells alone group on day 21 after allo-BMT, although the difference was not statistically significant on day 14 (Fig 5E).

Thus, our data suggest that combination therapy to treat aGVHD, with co-administration of MSCs and Treg cells, was effective for not only enhancing the maintenance of adoptive transferred Foxp3+ Treg cells, but also for increasing the amount of repopulated endogenous Treg cells in recipients.

### eGFP^+^ cells were detected at high levels in aGVHD target organs, especially in the combined cell therapy group

**Fig 6:**
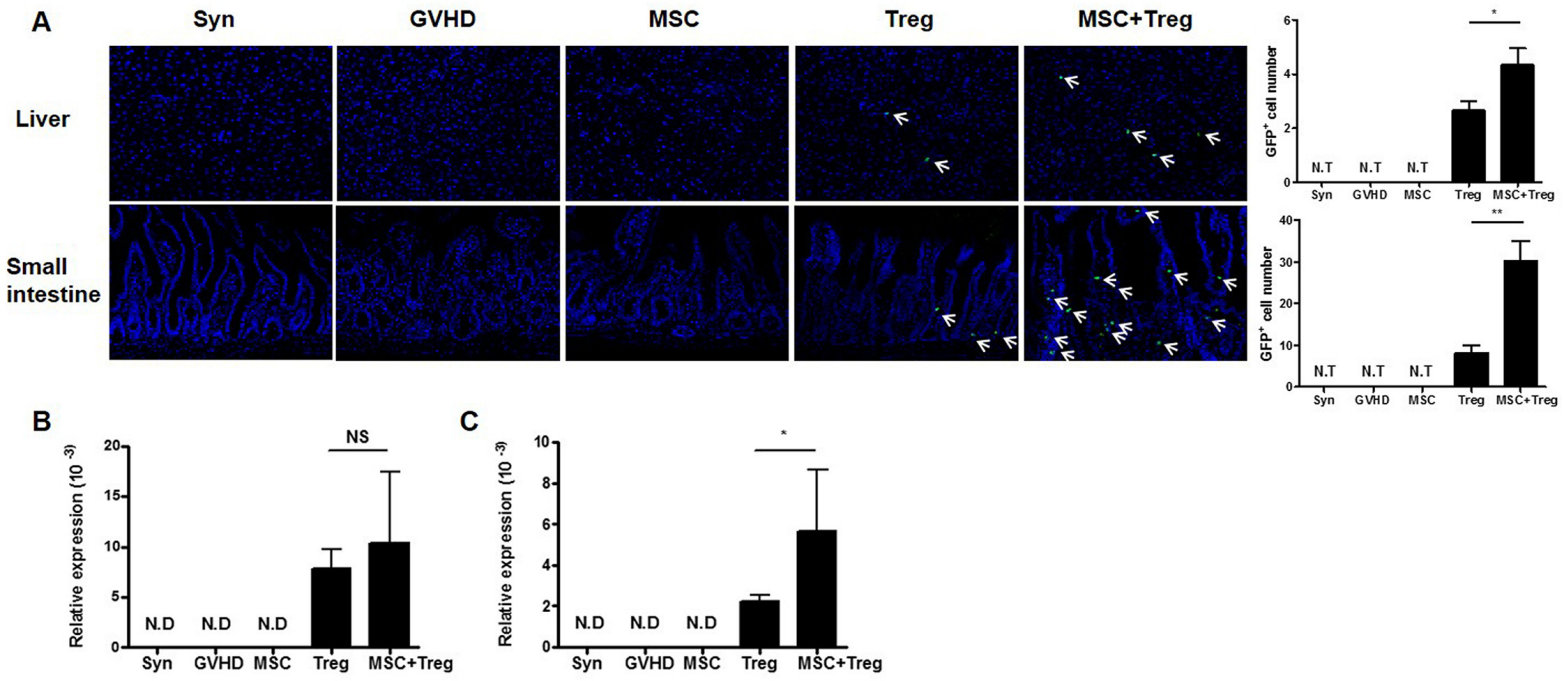
eGFP detection in aGVHD target organs. (A) Immunofluorescence staining with confocal examination illustrates eGFP^+^ cells in recipients. eGFP^+^ cells were counted in the liver and small intestine (right). (B–C) Expression of eGFP mRNA was examined by real-time PCR in recipient spleen and small intestine on day 7 after BMT (*n* = 6). Data are shown as the mean ± SEM. The results are representative of three independent experiments. *P* values were determined using Student’s *t*-test. * *p* < 0.05, ** *p* < 0.01.

We identified eGFP expression in the recipient liver and small intestine; aGVHD target organs were observed by immunofluorescence confocal microscopy. eGFP expression levels were detected in both the Treg cell group and the combined cell therapy group. However, the population of eGFP^+^ cells was higher in the combined cell therapy group than the Treg cells single group (Fig 6A). In addition, eGFP mRNA expression was examined in the recipient spleen and small intestine on day 7 after BMT. eGFP expression in both the spleen (Fig 6B) and small intestine (Fig 6C) was increased in the combined cell therapy group, but the difference was not statistically significant in the spleen. Thus, these results provide evidence for the ability of combination therapy to reduce aGVHD severity.

## Discussion

The population of Treg cells is an important factor in determining satisfactory therapeutic outcomes of aGVHD after allo-HSCT. For this reason, we expected that preserving the long-term survival of adoptively transferred Treg cells may provide an important strategy for preventing aGVHD without having them convert into other cells.

Several reports demonstrated that *in vitro* co-cultures of MSCs with T cells were able to induce Foxp3-expressing CD4^+^ T cells. This MSC-induced Foxp3^+^ Treg cell formation has also been demonstrated in several *in vivo* mouse models through MSC-mediated T cell suppression and the consequent induction of Treg cells [18‒22,30‒32]. For that reason, we considered that MSCs may be helpful in effectively maintaining and generating Treg cells in an aGVHD model via co-infusion of MSCs and Treg cells.

In contrast to our previous study, which used host-derived MSCs and Treg cells, we used donor-derived MSCs and Treg cells for treatment in this study. Our main objective was to distinguish between adoptively transferred donor-derived Treg cells and repopulating donor-derived endogenous Treg cells after BMT. The data suggest an underlying synergistic interaction between the combined cell therapy.

Numerous studies have shown that MSCs suppress GVHD in mice [33] and humans [34] by suppressing the proliferation of allo-reactive T cells [5]. However, our data show that MSCs alone had lower rates of survival and clinical scores. Recent data have shown that MSCs are influenced by environmental conditions, including inflammatory cytokines present during the early post-transplant period [35,36]. The immunosuppressive effects exhibited by MSCs are not constitutive, but are triggered by specific immune responses, including IFN-γ and TNF-α [32]. Thus, a lack of inflammatory cytokines in the model used in this study may have hindered the immunosuppressive effects of MSCs due to early administration on days 0 and 4 after BMT. Further studies are needed to determine the effects of MSC injection at different time points during the post-transplant period.

Several reports have shown that aGVHD is a Th1-mediated response in animal models [37]. Our results indicated that IFN-γ and TNF-α were downregulated in the combined cell therapy group versus MSCs or Treg cells alone, whereas IL-4 expression was not significantly different among the groups. The mRNA levels of T-bet were slightly lower in the combined cell therapy group, while GATA-3 was not significantly different among groups. No groups had significantly different levels of T-bet or GATA3. In addition, STAT1 expression was significantly downregulated in the combination cell therapy groups. These results suggest that combined cell therapy with MSCs and Treg cells was associated with downregulation of the Th1 response in the aGVHD model. However, MSCs alone had increased levels of IFN-γ compared to the GVHD group, rather than an inhibitory effect. Previous studies showed that MSCs alone do not inhibit the development of the inflammatory cytokines involved in autoimmune diseases, including joint inflammation and autoimmune arthritis [14,38]. In fact, recent reports suggest that the administration of MSCs can aggravate inflammatory responses [39]. Thus, MSCs alone may not have been sufficient for effective downregulation of inflammatory cytokines in this study.

According to Bettelli et al. [34], conversion of Treg cells is caused by IL-6, which acts as a potent inflammatory cytokine, promoting Th17 development, and inhibiting the generation of Treg cells. We examined Th17 cells to identify the possibility of the conversion of Treg cells in recipient spleen and blood. The percentage of IL-17^+^ cells in CD4^+^ T cells was significantly decreased in the combined cell therapy group at 12 days after BMT. Although IL-6 expression in CD4^+^ T cells was scarcely detected in any group, there was further downregulation in the combined cell therapy group. Likewise, STAT3 and RORγ-t expression was significantly decreased in the combined cell therapy group.

These data suggest that combined cell therapy can influence the downregulation of Th17 cells in aGVHD. Although we used induced Treg cells (iTreg), which are more resistant to converting into Th17 cells than natural Treg cells (nTreg) [40], according to Koenecke et al. [36], even adoptively transferred iTreg cells are unstable both *in vitro* and *in vivo*, lacking the suppressed activity to prevent fatal aGVHD. Thus, we examined the conversion of Treg cells using *ex vivo*-expanded Treg^egfp^ cells of high purity (> 96%), and co-administered with MSCs after BMT. Then, we observed IL-17 cells in CD4^+^ T cells to see whether adoptively transferred Treg^egfp^ cells were converted into Th17 cells. As a result, CD4^+^ IL-17^+^ cells were detected at < 5% of CD4^+^ T cells in the spleen and blood at day 21, and eGFP^+^ cells were detected as a small proportion of CD4^+^ IL-17^+^ cells. In addition, CD4^+^ IL-17^+^ cells were decreased in the combined cell therapy group on day 21 versus the Treg cells group. Thus, few of our adoptively transferred Treg^egfp^ cells were converted into Th17 cells in the aGVHD model. Indeed, our results suggest that combined cell therapy using MSCs and Treg cells was effective at reducing the conversion of CD4^+^ T cell into Th17 cells.

Our previous studies suggested that co-treatment with host-derived MSCs and Treg cells increased Foxp3^+^ Treg cells in aGVHD recipients [23]. In the present study, CD4^+^ CD25^+^ cells were increased in the groups that received cells for therapy, except the GVHD group. However, Foxp3^+^ cells were only effectively increased in the combined cell therapy group. SOCS3 and Foxp3 expression at the transcriptional level were also increased greatly in the combined cell therapy group. Thus, co-administration of MSCs and Treg cells could offer benefits for the treatment of aGVHD [41]. Following these results, we focused on the increase in Foxp3^+^ Treg cells in recipients using *ex vivo*-expanded Treg^egfp^ cells to distinguish between adoptively transferred Treg cells and endogenously generated Treg cells. As a result, adoptively transferred Treg^egfp^ cells were detected at high levels in the spleen and blood in both the Treg cell group and the combined cell therapy group in the early transplant period. Treg^egfp^ cells were similarly decreased in both groups by day 14. However, Treg^egfp^ cells were only significantly preserved in the combined cell therapy group, compared to the Treg cells single group, on day 21. Furthermore, the Treg cells (H-2^b^) generated endogenously from recipients were significantly increased in the combined cell therapy group on day 21. Together, these results indicate that MSCs not only influenced the long-term survival of transferred Treg^egfp^ cells, but also induced the repopulation of endogenous Treg cells in the recipient after BMT.

aGVHD is a major complication of allogeneic hematopoietic stem cell transplantation (allo-HSCT), which leads to tissue damage, including the gut, skin, liver, and lungs [42,43]. We identified improvements in the histological examination through combined cell therapy in aGVHD target organs. We also examined eGFP expression in the liver and small intestine by immunofluorescence staining at 7 days after BMT. eGFP expression was detected more strongly in the combined cell therapy group than in the Treg cells single group. Moreover, eGFP mRNA expression levels in the spleen and small intestine were increased in the combined cell therapy group on day 7 after BMT. These results suggest that the adoptive transferred Treg cells influenced the aGVHD target organs, and that MSCs may contribute to maintaining the survival of transferred Treg cells in aGVHD target organs through co-infusion of MSCs and Treg cells after BMT.

In summary, the present study suggests that combined cell therapy with donor-derived MSCs and Treg cells improved the survival and manifestation of aGVHD, and downregulated Th1 and Th17 responses in recipients. Moreover, MSCs induced the long-term survival of transferred Treg cells, particularly in aGVHD target organs, and increased the repopulation of endogenous Treg cells in recipients after BMT. These data demonstrate a potential strategy of combined cell therapy with MSCs and Treg cells for preventing aGVHD.

## Supporting Information

S1 Checklist ARRIVE Guidelines Checklist 2014. (DOC)
