# Supplementary material for: Adoptive Transfer of Treg Cells Combined with Mesenchymal Stem Cells Facilitates Repopulation of Endogenous Treg Cells in a Murine Acute GVHD Model
Source: PLoS One. 2015 Sep 22;10(9):e0138846. doi: 10.1371/journal.pone.0138846 (PMC4578951; doi:10.1371/journal.pone.0138846)

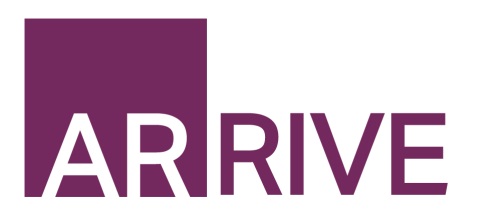


The ARRIVE Guidelines Checklist

Animal Research: Reporting In Vivo Experiments

Carol Kilkenny^1^, William J Browne^2^, Innes C Cuthill^3^, Michael Emerson^4^ and Douglas G Altman^5^

*^1^The National Centre for the Replacement, Refinement and Reduction of Animals in Research, London, UK, ^2^School of Veterinary Science, University of Bristol, Bristol, UK, ^3^School of Biological Sciences, University of Bristol, Bristol, UK, ^4^National Heart and Lung Institute, Imperial College London, UK, ^5^Centre for Statistics in Medicine, University of Oxford, Oxford, UK.*

|  | | ITEM | RECOMMENDATION | Section/ Paragraph |
| --- | --- | --- | --- | --- |
|  | 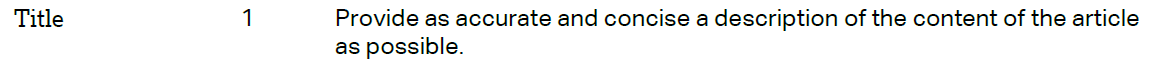 | | | Title |
|  | 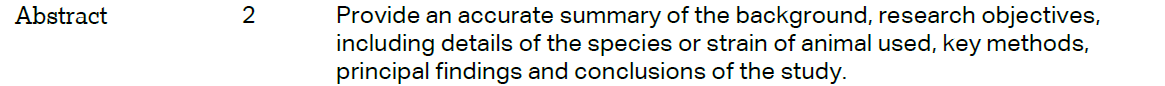 | | | Abstract/1 |
|  | INTRODUCTION | | |  |
|  | 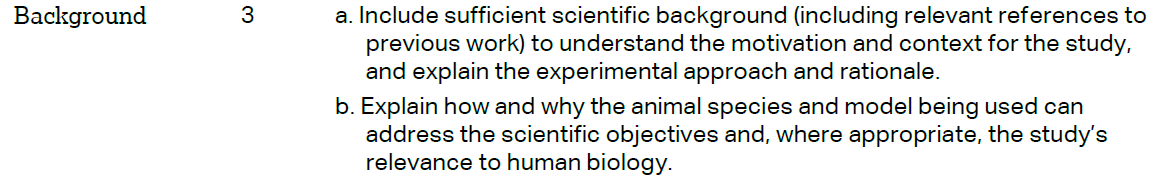 | | | Introduction/1,4 |
|  | 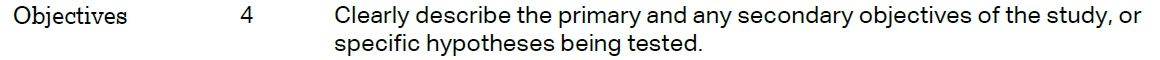 | | | Introduction/2 |
|  | METHODS | | |  |
|  | 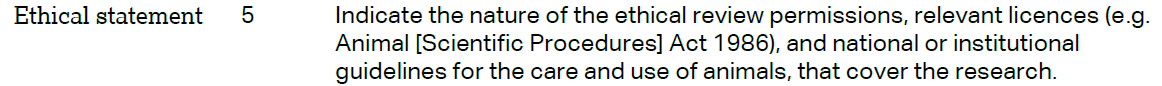 | | | Materials and Methods /1 |
|  | 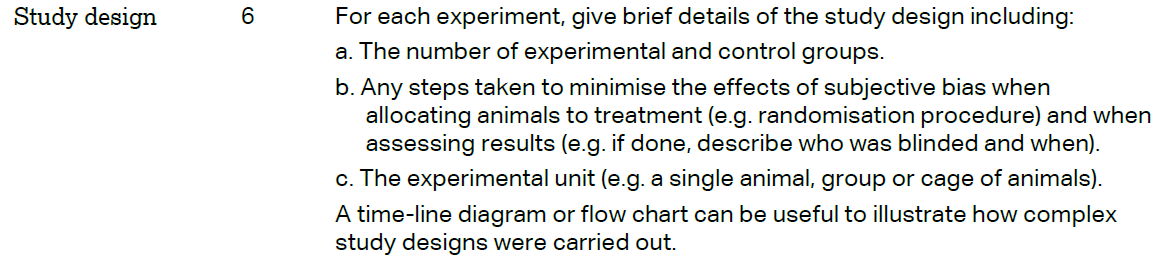 | | | Materials and Methods /4 |
|  | 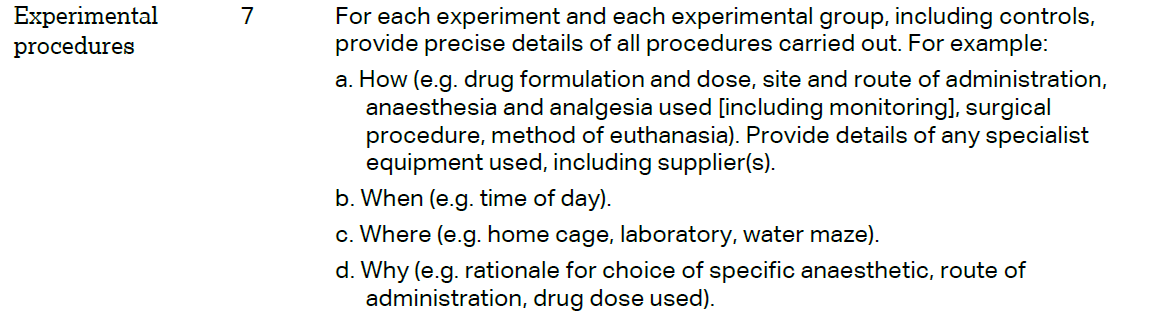 | | | Materials and Methods /4,5 |
|  | 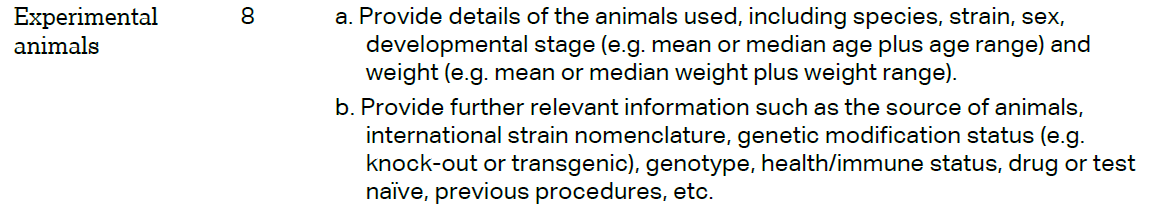 | | | Materials and Methods /1 |

The ARRIVE guidelines. Originally published in *PLoS Biology*, June 2010^1^

|  | 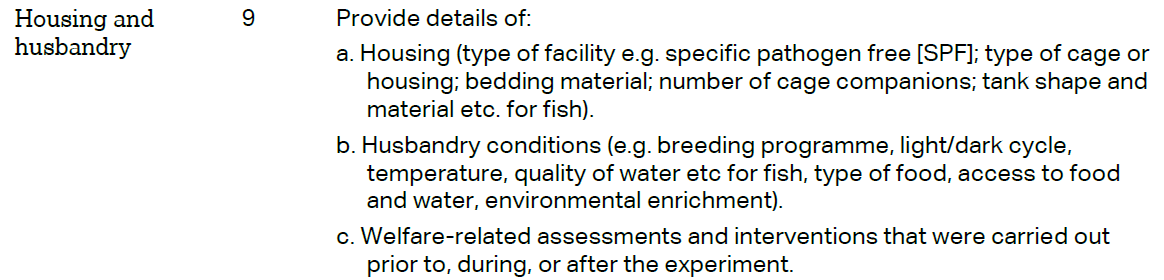 | Materials and Methods /1 | |
| --- | --- | --- | --- |
|  | 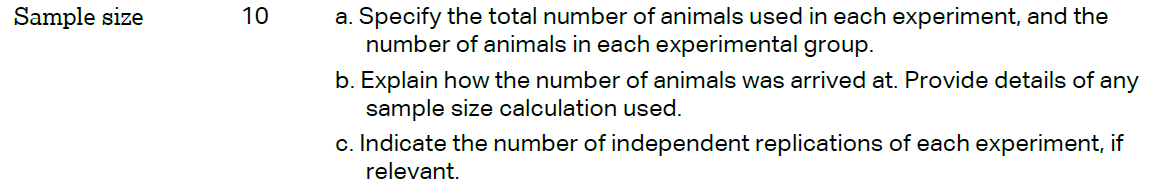 | Materials and Methods/1,  Figure legends/2 | |
|  | 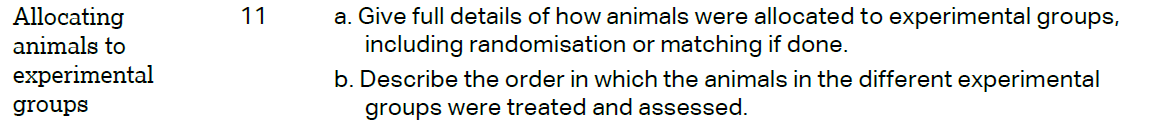 | Materials and Methods /1 | |
|  | 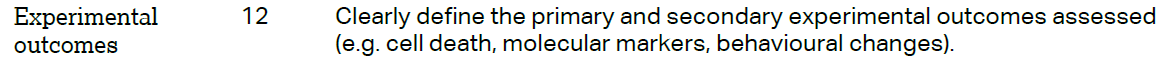 | Materials and Methods /2, 3 | |
|  | 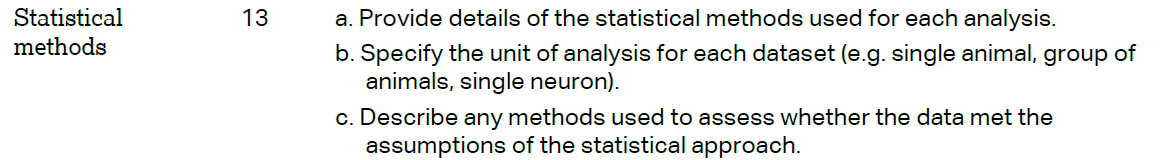 | Materials and Methods/11 | |
|  | RESULTS |  | |
|  | 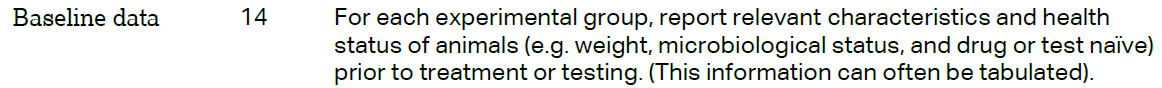 | Results/2 | |
|  | 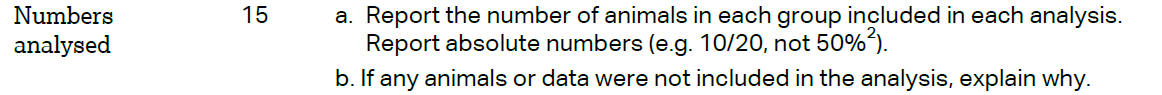 | Figure legends/2 | |
|  | 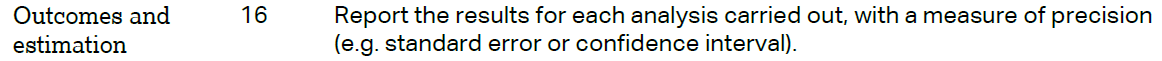 | Figure legends/2 | |
|  | 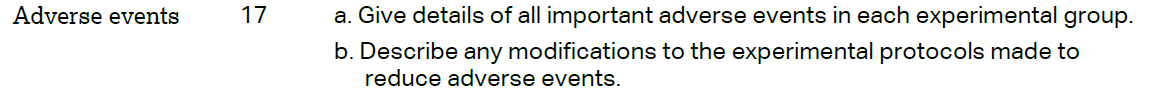 | Results/2 | |
|  | DISCUSSION |  | |
|  | 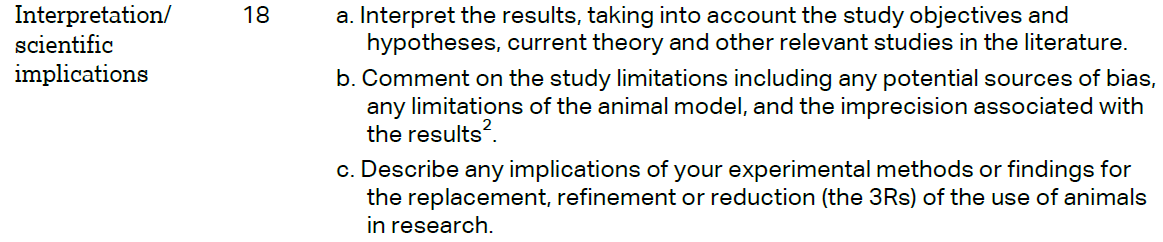 | Discussion/1 | |
|  | 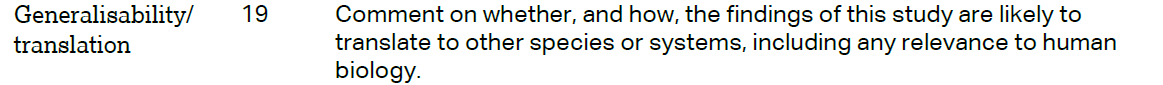 | Discussion /7 | |
| 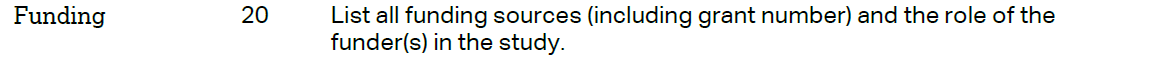 | | Acknowledgments/1 |  |


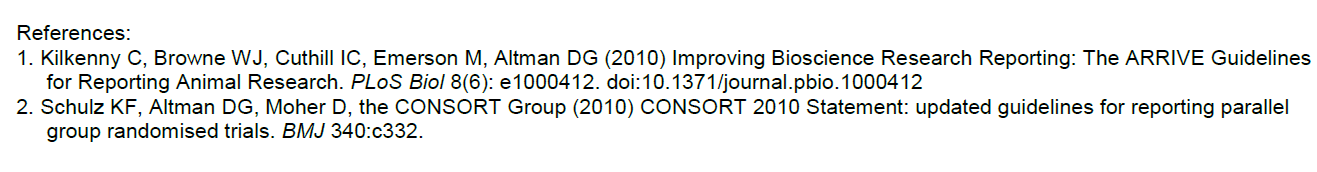

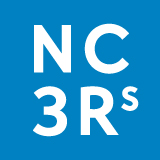

Supplement: S1 Checklist — (DOC) [file pone.0138846.s001.doc]
